# Huge right ventricular mass lesion associated with genital malignant tumor: a case report

**DOI:** 10.1186/s13256-017-1439-4

**Published:** 2017-10-03

**Authors:** Masaya Higashi, Akihiko Hodatsu, Katsuharu Uchiyama, Hayato Tada, Mika Mori, Miho Ohira, Kenshi Hayashi, Masa-aki Kawashiri

**Affiliations:** 1 Division of Cardiology, Keiju General Hospital, Nanao, Japan; 2 Department of Cardiovascular and Internal Medicine, Kanazawa University Graduate School of Medicine, 13-1 Takara-machi, Kanazawa, 920-8641 Japan

**Keywords:** Cardiac tumor, Metastatic heart tumor, Congestive heart failure

## Abstract

**Background:**

Primary heart tumors are rare, whereas metastatic heart tumors occur more frequently.

**Case presentation:**

We report a case of a 75-year-old Japanese woman who had metastatic heart tumors of the right ventricle. Although she initially received antibiotic therapy following a diagnosis of pneumonia and pleuritis, her symptoms worsened, and she developed dyspnea and bilateral lower limb edema. Echocardiography showed a huge mass lesion occupying the entire right ventricle. Because the patient’s tumor markers were elevated, we used computed tomography to search for the primary lesion, which was located in the vagina or the uterus. Histology demonstrated the presence of basaloid squamous cell carcinoma in the vaginal tissue. Chemotherapy with paclitaxel and carboplatin was initiated.

**Conclusions:**

These data suggest that the tumor in the right ventricle metastasized from the genital organs.

## Background

Primary heart tumors are rare, appearing in 0.02% of autopsies [1]. Metastatic heart tumors occur more frequently, and their probability of occurrence ranges from 2.3% to 18.3% [2]. In general, metastatic heart tumors are asymptomatic. Although these metastases rarely grow in the heart cavity, they often lead to lethal complications, such as embolism and obstruction of heart cavity inflow or outflow. We report a case of a patient with multiple metastases from the genital organs, including the right ventricle.

## Case presentation

A 75-year-old Japanese woman was admitted to our clinic because of dyspnea associated with bilateral lower limb edema. She was well until 8 weeks before admission, when she began to complain of back pain and cough. Although a local doctor gave her antibiotics after a diagnosis of pneumonia, her symptoms worsened. One week later, she consulted our clinic, where different antibiotics were prescribed to relieve her symptoms. However, she complained of dyspnea with bilateral lower limb edema, which suggested congestive heart failure.

**Fig. 1:**
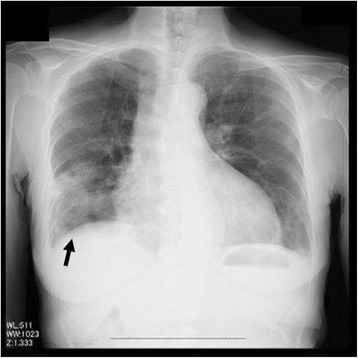
Chest x-ray showing enlarged heart associated with infiltration of both lung fields, particularly in the lower right area (*black arrow*)

The patient’s oxygen saturation on admission was 90%, although her blood pressure (124/82 mmHg) and heart rate (95 beats/minute) were stable. Coarse crackles were audible in the lung field. Significant bilateral lower limb edema was observed. Blood analysis revealed that the patient had a C-reactive protein level of 7.53 mg/dl and a brain-type natriuretic peptide level of 668.5 pg/ml. Although the patient’s electrocardiogram (ECG) did not produce any significant findings, her chest x-ray showed an enlarged heart associated with infiltration of both lung fields, particularly in the lower right area (Fig. 1).

**Fig. 2:**
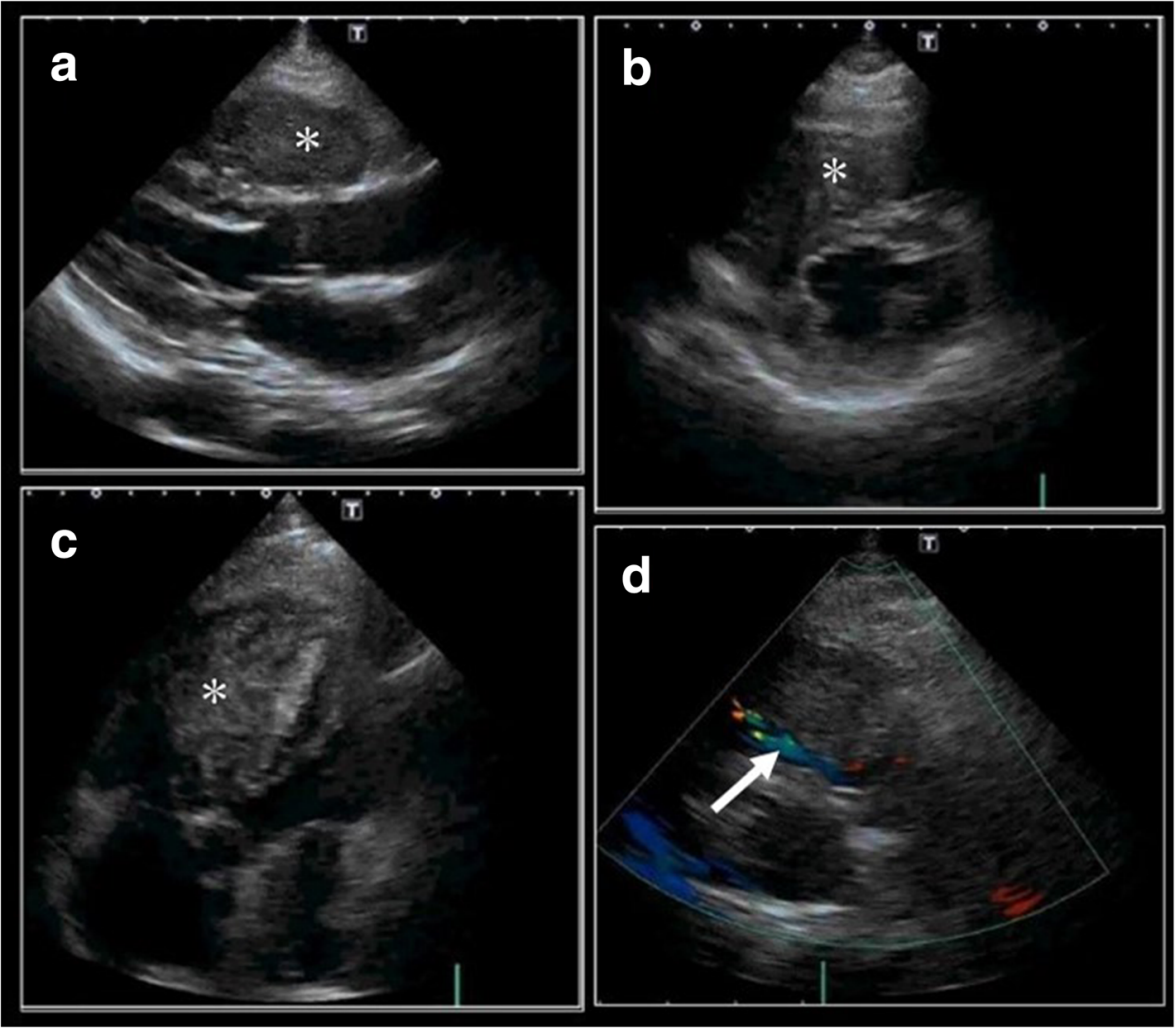
Echocardiograms. **a**–**c** Echocardiograms revealing a huge mass occupying the right ventricle (*). There were no masses in the right atrium. The patient’s right ventricular pressure was elevated, and the interventricular septum was flattened. **d** Color Doppler echocardiogram showing a disturbance in right ventricular outflow (*white arrow*)

**Fig. 3:**
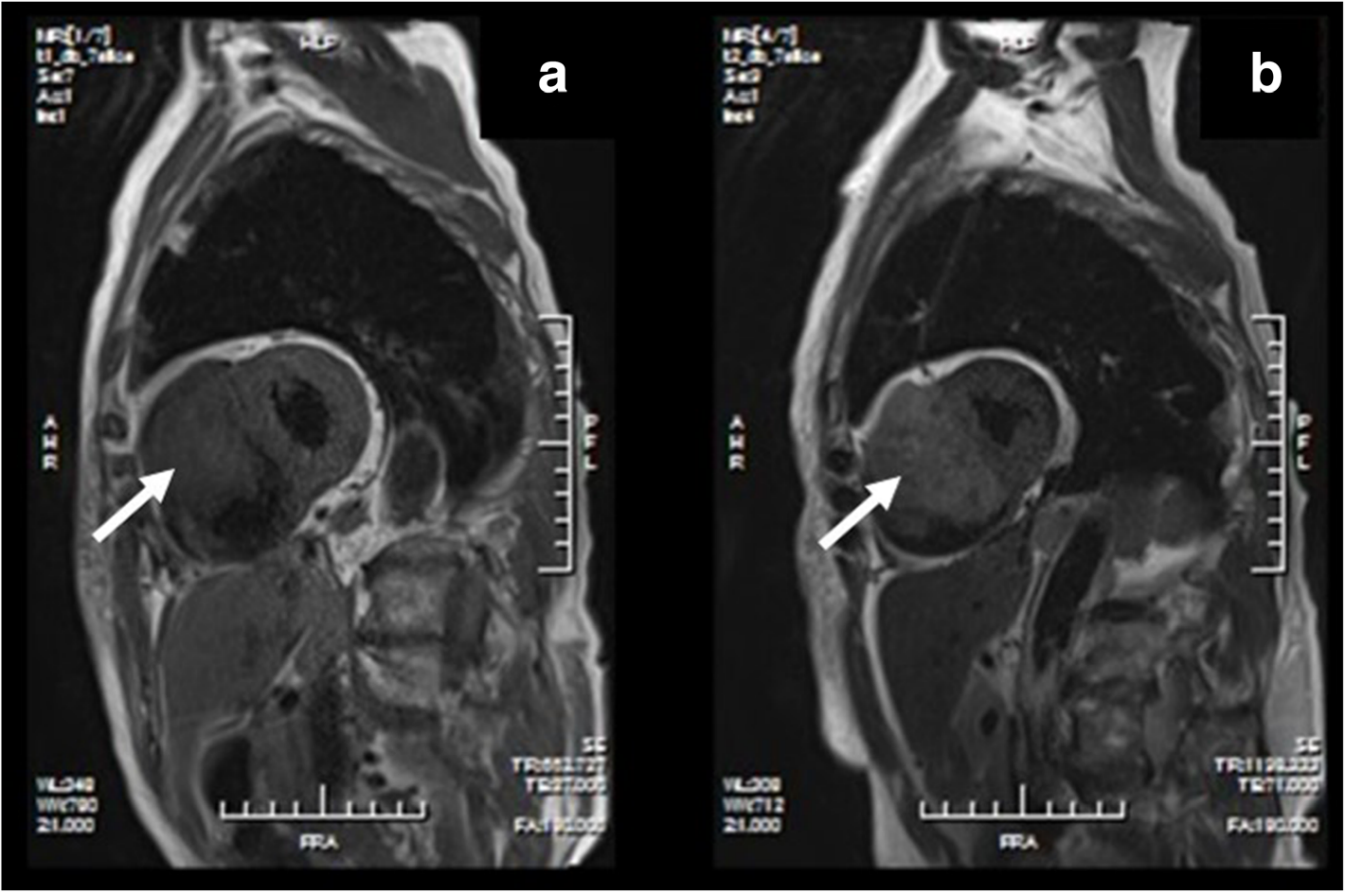
Cardiac magnetic resonance imaging studies. **a** and **b** The presence of the tumor was confirmed by cardiac magnetic resonance imaging, which showed a mass occupying all the space in the right ventricle. The tumor and interventricular septum were poorly marginated. The *arrows* indicate tumors

When we examined the patient’s echocardiogram, we saw a huge mass occupying the entire right ventricle. However, there was no mass lesion in the right atrium, suggesting the possible occurrence of the original tumor in the right ventricle (Fig. 2). Owing to obstruction of the right ventricle, the calculated right ventricular pressure was 49.5 mmHg. The right ventricular mass was confirmed by cardiac magnetic resonance imaging, which showed a mass occupying the entire right ventricle (Fig. 3). Importantly, the patient’s cancer antigen 125 (CA 125) and soluble interleukin 2 receptor (sIL-2R) levels were markedly elevated at 135.6 U/ml and 672 U/ml, respectively.

**Fig. 4:**
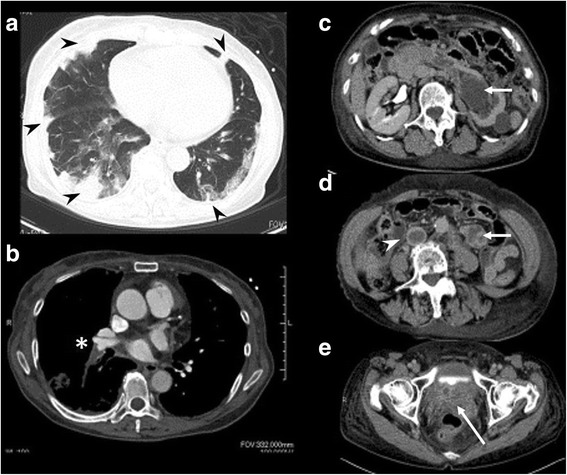
Whole-body computed tomographic images showing several metastatic lesions. The *arrowheads* and *asterisk* indicate tumors. **a** Multiple nodular and infiltrative shadows were seen mainly in the peripheral lung field. **b** Contrast-enhanced computed tomography revealed tumor emboli in the right pulmonary artery. **c**, **d** Left hydronephrosis indicates involvement of the lymph node near the left urinary duct. There were tumor thrombi in the inferior vena cava leading to the bilateral common iliac vein. **e** The uterus was completely occupied by the tumor﻿

**Fig. 5:**
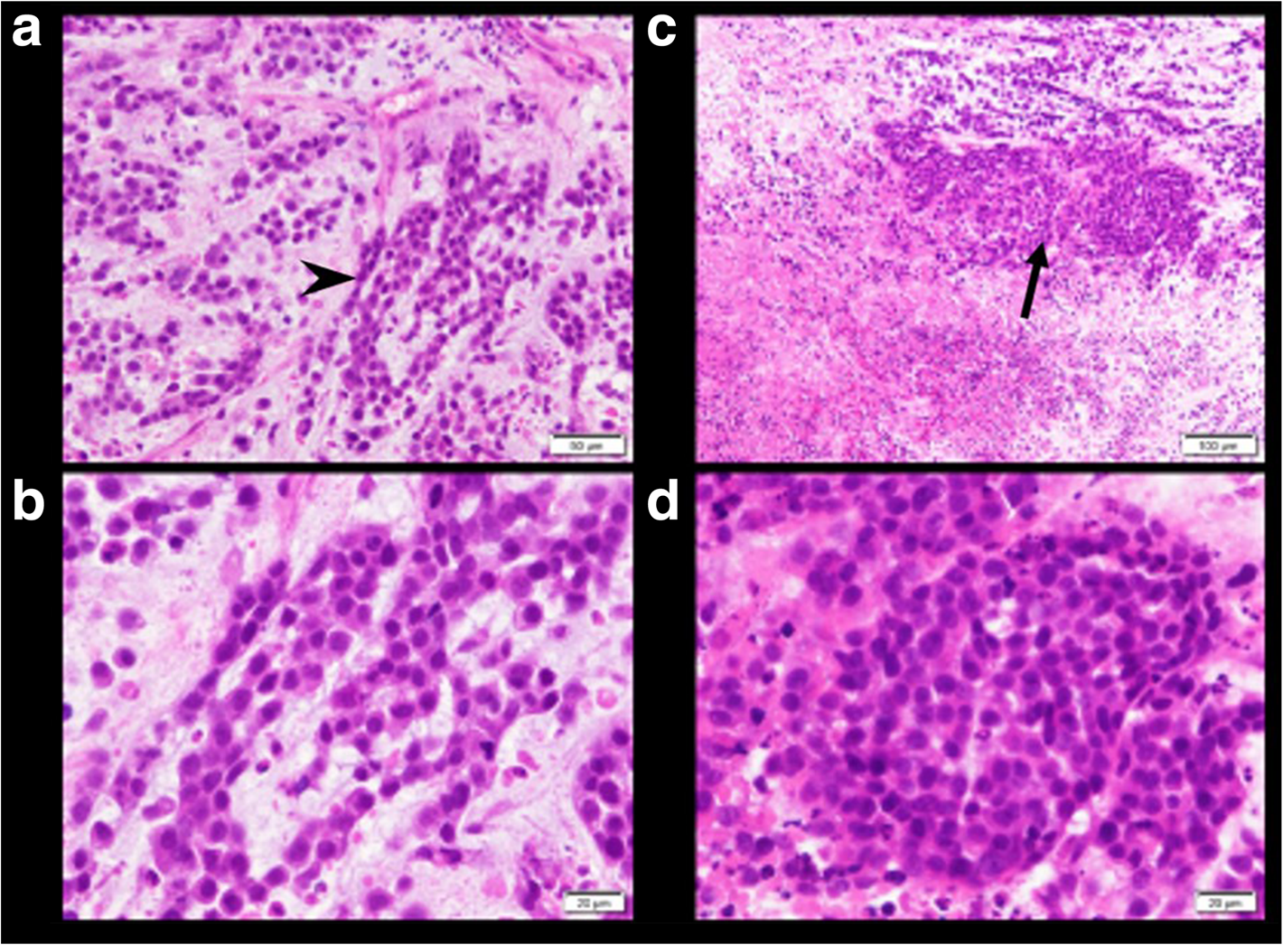
Histological studies showing that large numbers of small basaloid cells (*arrow﻿head*)﻿ had invaded and displayed cordlike (**a**, **b**) or alveolar structures (**c**, **d**). The *arrows* indicate tumors

Because of the elevation of tumor markers, such as CA 125 and sIL-2R, we carefully searched for malignant lesions. In addition to pulmonary embolization, there were several metastatic lesions in the lung field, pleura, inferior vena cava, lymph nodes, and kidney (Fig. 4). Importantly, the original tumor was located in the uterus and vagina, where it occupied the entire space (Fig. 4). Histology demonstrated basaloid squamous cell carcinoma in the vaginal tissue (Fig. 5), and chemotherapy with paclitaxel (120 mg) and carboplatin (340 mg) according to the protocol for cancer of the uterine cervix was initiated, although the effectiveness of chemotherapy on cardiac metastasis is unclear.

## Discussion

Most cases of cardiac metastatic tumor are asymptomatic until the tumor obstructs the heart chamber. In that situation, systemic chemotherapy has priority over resection of the tumor because surgical resection is indicated only for exceptional cases [3]. If complete resection fails, postoperative mortality is high [4].

Basaloid squamous cell carcinoma is a rare and highly aggressive variant of squamous cell carcinoma [5]. The most commonly involved sites are the larynx, hypopharynx, tonsils, and base of the tongue, not the heart [6]. In our patient, the squamous cell carcinoma antigen was not elevated, although it is a variant of squamous cell carcinoma [7], as shown in other cases [8, 9]. Accordingly, in the case of our patient, it was difficult to diagnose the primary lesion on the basis of blood markers.

However, most tumors in the right side of the heart are metastatic malignant tumors [10], although some primary cardiac tumors have been reported [11]. Our patient initially developed respiratory problems that were followed by congestive heart failure. Clinically, cardiac metastasis usually remains asymptomatic. However, echocardiography should be performed as soon as symptoms of heart failure, angina pectoris, embolism, or rhythm disturbances develop; if a new heart murmur becomes audible; or if heart size is increased on a chest x-ray. Alternatively, additional information may be obtained by computed tomography or magnetic resonance imaging [12].

## Conclusions

We suggest that clinicians carefully examine the heart in patients with malignant tumors of the genital organs.

## References

[1] Reynen K. Frequency of primary tumors of the heart. Am J Cardiol. 1996;77:107. 10.1016/s0002-9149(97)89149-7 8540447

[2] Bussani R, De-Giorgio F, Abbate A, Silvestri F. Cardiac metastases. J Clin Pathol. 2007;60:27–34. 10.1136/jcp.2005.035105 PMC1860601 17098886

[3] Gibbs P, Cebon JS, Calafiore P, Robinson WA. Cardiac metastases from malignant melanoma. Cancer. 1999;85:78–84. 9921977

[4] Poole GV, Meredith JW, Breyer RH, Mills SA. Surgical implications in malignant cardiac disease. Ann Thorac Surg. 1983;36:484–91. 10.1016/s0003-4975(10)60494-8 6354118

[5] Martínez-Girón R, Martínez-Torre S, Mosquera-Martínez AJ. Basaloid squamous cell carcinoma of the uterine cervix: cytological and histological features. Diagn Cytopathol. 2015;43:993–5. 10.1002/dc.23364 26391899

[6] Vasudev P, Boutross-Tadross O, Radhi J. Basaloid squamous cell carcinoma: two case reports. Cases J. 2009;2:9351. 10.1186/1757-1626-2-9351 PMC2804002 20062602

[7] Gadducci A, Tana R, Cosio S, Genazzani AR. The serum assay of tumour markers in the prognostic evaluation, treatment monitoring and follow-up of patients with cervical cancer: a review of the literature. Crit Rev Oncol Hematol. 2008;66:10–20. 10.1016/j.critrevonc.2007.09.002 17964182

[8] Kawaguchi A, Shibata J, Naito H, Endo Y, Kodama M. One case of basaloid carcinoma of the esophagus [in Japanese]. Nihon Shokaki Geka Gakkai Zasshi. 1994;27:892–6.

[9] Maruyama T, Endo M, Hirayama N, Murakami K, Matsubara H, Shioda K. A resected case of metastasis to the small intestine from esophageal basaloid cell carcinoma presented with small bowel obstruction. J Japan Surg Assoc. 2011;72:333–8.

[10] Yuda S, Nakatani S, Yutani C, Yamagishi M, Kitamura S, Miyatake K. Trends in the clinical and morphological characteristics of cardiac myxoma: 20-year experience of a single tertiary referral center in Japan. Circ J. 2002;66:1008–13. 10.1253/circj.66.1008 12419931

[11] Yamagishi M, Bando K, Furuichi S, Ishibashi-Ueda H, Yutani C, Miyatake K. Images in cardiovascular medicine: primary cardiac osteosarcoma in right ventricular outflow tract. Circulation. 2000;101:2220–1. 10.1161/01.cir.101.18.2220 10801765

[12] Reynen K, Köckeritz U, Strasser RH. Metastases to the heart. Ann Oncol. 2004;15:375–81. 10.1093/annonc/mdh086 14998838

